# Oncolytic adenovirus-mediated expression of decorin facilitates CAIX-targeting CAR-T therapy against renal cell carcinoma

**DOI:** 10.1016/j.omto.2021.11.018

**Published:** 2021-11-29

**Authors:** Chen Zhang, Lin Fang, Xueyan Wang, Sen Yuan, Wanjing Li, Weiping Tian, Jing Chen, Qi Zhang, Yuxin Zhang, Qing Zhang, Junnian Zheng

**Affiliations:** 1 Jiangsu Key Laboratory of Biological Cancer Therapy, Cancer Institute, Xuzhou Medical University, 84 West Huai-hai Road, Xuzhou 221002, Jiangsu, China; 2 Department of Oncology, The First People's Hospital of Yancheng, Yancheng 224001 Jiangsu, China; 3 Center of Clinical Oncology, Affiliated Hospital of Xuzhou Medical University, Xuzhou 221002, China

**Keywords:** oncolytic adenovirus, chimeric antigen receptor T cell, Decorin, carbonic anhydrase IX, renal cell carcinoma

## Abstract

Although chimeric antigen receptor T cell (CAR-T) therapy has been successful for hematological malignancies, it is less effective for solid tumors. The primary reason is that the immune microenvironment restricts CAR-T cells from infiltrating and proliferating in tumors. Oncolytic virotherapy has emerged as a novel immunogenic therapy to augment antitumor immune response. Here we combined an oncolytic adenovirus carrying decorin with a CAR-T targeting carbonic anhydrase IX (CAIX) to perform the antitumor activity for renal cancer cells. We found that OAV-Decorin combined with CAIX-CAR-T exhibited significantly reduced tumor burden, altered the composition of extracellular matrix (ECM) by inhibiting the distribution of collagen fibers, decreased the expression of TGF-β in tumor cells, enhanced IFN-γ secretion, and obtained higher numbers of CAR-T cells. The combination treatment modality showed prolonged mice survival. The intratumoral injection of OAV-Decorin into tumor-bearing immunocompetent mice activated the inflammatory immune status and resulted in tumor regression. These data supported further investigation of the combination of OAV-Decorin and CAIX-CAR-T cells in solid tumors.

## Graphical abstract

**Figure:**
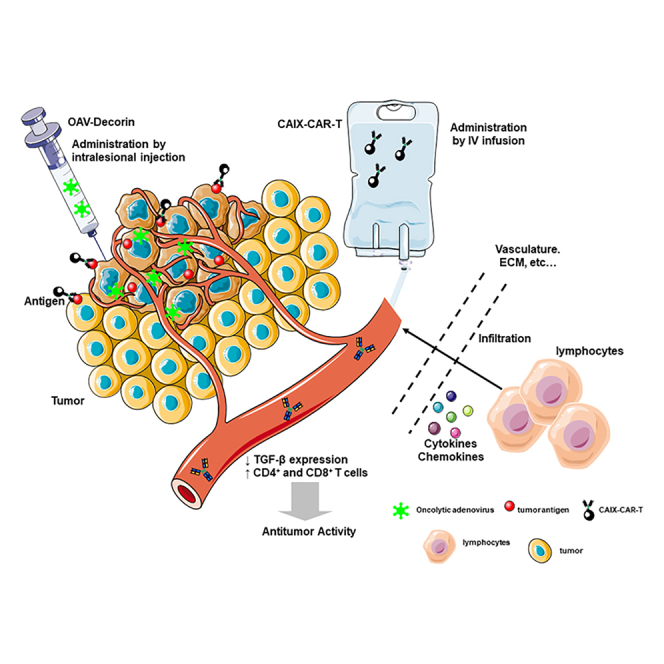

## Teaser

It was shown that decorin expression mediated by oncolytic adenovirus activated the inflammatory immune status. OAV-Decorin combined with CAIX-targeted CAR-T cells exhibited significantly reduced renal tumor burden and altered the composition of extracellular matrix. OAV-Decorin is potent to be developed as an effective enhancer of CAR-T cells therapeutic approach.

## Introduction

Renal cell carcinoma (RCC) is one of the most common tumors in the urinary system.^1^^,^^2^ Recently many research papers support that the tumor microenvironment (TME) plays a critical role in tumor development and progression.^3^^,^^4^ Various factors among TME can produce physical and immune barriers to the host immune system. Therefore, there is an urgent need to overcome these barriers to improve antitumor activity.

Immunotherapies have provided remarkable clinical benefit to patients with different types of cancer.^5^^,^^6^ Chimeric antigen receptor T cell (CAR-T) is a landmark achievement of tumor immunotherapy in recent years.^7^^,^^8^ Chimeric antigen receptor (CAR) is an artificial receptor that mimics the function of a T cell receptor (TCR). It artificially integrates TCR binding signals, CD28 and CD137 co-stimulatory signals required for T cell activation into a single signal receptor composed of target recognition domains, hinge and transmembrane domains, and intracellular signal domains.^9^ CAIX (carbonic anhydrase IX), also known as G250, is a transmembrane glycoprotein that is highly expressed in renal cancer cells.^10^ Weijtens et al. constructed a CAR composed of scFv (variable fragment of single chain antibody) of mouse anti-human CAIX in tandem with FCRγ, and carried out *in vitro* CAR-T experiments.^11^ Lamers et al. used CAIX CAR-T in a phase I/II clinical trial for renal cancer, and the results showed that plasma levels of interferon (IFN)-γ, interleukin (IL)-2, and tumor necrosis factor-α increased in most patients, but no clinical efficacy was observed.^12^ There were some factors, such as the tumor immunosuppressive microenvironment, that limited the effectiveness of the CAR-T cells.

Oncolytic virus (OV) selectively replicates and lyses tumor cells after genetic engineering.^13^ It has emerged as alternative immune therapeutic approach due to the potential effects on producing immune activation.^14^^,^^15^ Decorin is a low molecular proteoglycan rich in leucine that is widely distributed in tissues and can bind to a variety of growth factors and cytokines to regulate cell growth and tissue remodeling.^16^^,^^17^ Decorin can bind and inhibit transforming growth factor β (TGF-β) signaling.^18^, ^19^, ^20^ Oncolytic adenovirus-mediated-decorin expression significantly inhibited tumor growth and metastasis in various cancer models.^21^^,^^22^ However, immune tolerance was also always induced after oncolytic adenovirus therapy.^23^^,^^24^

In order to improve the efficacy of CAR-T and oncolytic adenovirus in tumor immunosuppressive microenvironment, here we used an oncolytic adenovirus arming decorin (OAV-Decorin) in combination with CAIX-targeting CAR-T for renal cancer cells. In our evaluation, tumor cells were lysed directly by OAV-Decorin to recruit CAR-T to the tumor site. On the other hand, decorin carried by OAV improved the permeability of tumor tissues and changed the tumor immunosuppressive microenvironment by boosting the inflammatory immune status, so that CAR-T or lymphocytes could infiltrate into tumor tissues more and played a better antitumor affect. These findings provided strategies for overcoming tumors resistant to immunotherapies and a rationale for further evaluation in humans.

## Results

### Characterization of an armed oncolytic adenovirus expressing decorin *in vitro*

**Figure 1:**
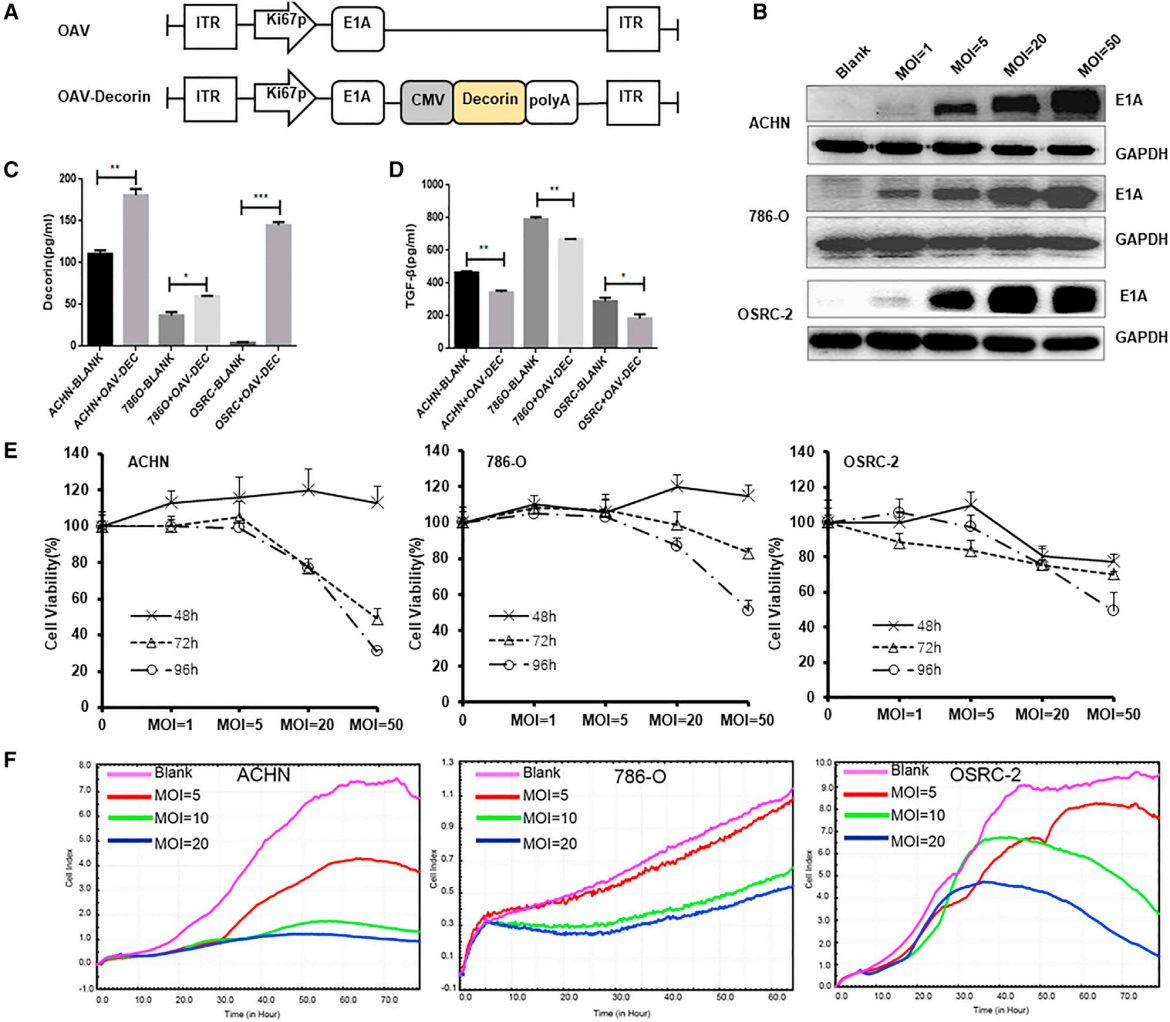
The armed oncolytic adenovirus expressing decorin (OAV-Decorin) successfully inhibits renal cancer cell (RCC) growth *in vitro* (A) Schematic representation of genome of the oncolytic adenoviruses used in this study. (B) ACHN, 786O, and OSRC-2 cells were infected with OAV-DEC at the indicated MOIs; 48 h later, the key adenoviral E1A protein was confirmed by Western blot. GAPDH was used as a loading control. (C, D) Supernatants were obtained 48 h post infection by OAV-DEC (referred to OAV-Decorin), and the concentration of decorin and TGF-β were analyzed by ELISA assay. (E) ACHN, 786O, and OSRC-2 cells were infected with OAV-DEC at a various MOIs (1, 5, 20, and 50) for 48, 72, or 96 h, respectively. Cell viability was measured using the CCK-8 assay. Relative cell viability was calculated compared with the control group, which was set at 1. (F) ACHN, 786O, and OSRC-2 cells were infected with OAV-DEC at various MOIs (5, 20, and 50). Viable cells were calculated as “cell index” using the xCELLigence RTCA system. ∗p < 0.05, ∗∗p < 0.01, ∗∗∗p < 0.001.

We generated an engineered oncolytic adenovirus expressing decorin named OAV-Decorin (OAV-DEC) (Figure 1A), in the backbone of a tumor-selective oncolytic adenovirus ZD55 vector, in which E1B-55KD had been deleted. To assess related gene expression mediated by the OAV-DEC, renal cancer cells 786O, ACHN, and OSRC-2 were treated with OAV-DEC at multiplicity of infection (MOI) 1, 5, 20, or 50. The adenoviral E1A expression level was dose-dependently elevated in three cancer cells (Figure 1B). Compared with the corresponding control groups, higher level of decorin was produced and efficiently released from OAV-DEC-infected renal cancer cells, as detected by ELISA (Figure 1C). Moreover, the TGF-β was also examined in the infected renal cancer cells. As shown in Figure 1D, TGF-β expression was lower in these cancer cells treated with OAV-DEC. The therapeutic efficacy of the OAV-DEC for different renal cancer cells was further quantified by CCK8 and real-time cell analysis (RTCA) results after infecting the cell lines at different MOI (Figures 1E and 1F). These results demonstrated that the armed oncolytic adenovirus OAV-DEC can efficiently infect renal cancer cells to produce and secrete high level of functional decorin.

### CAIX-CAR-T exerted selective cytotoxicity to CAIX-positive renal cancer cells

**Figure 2:**
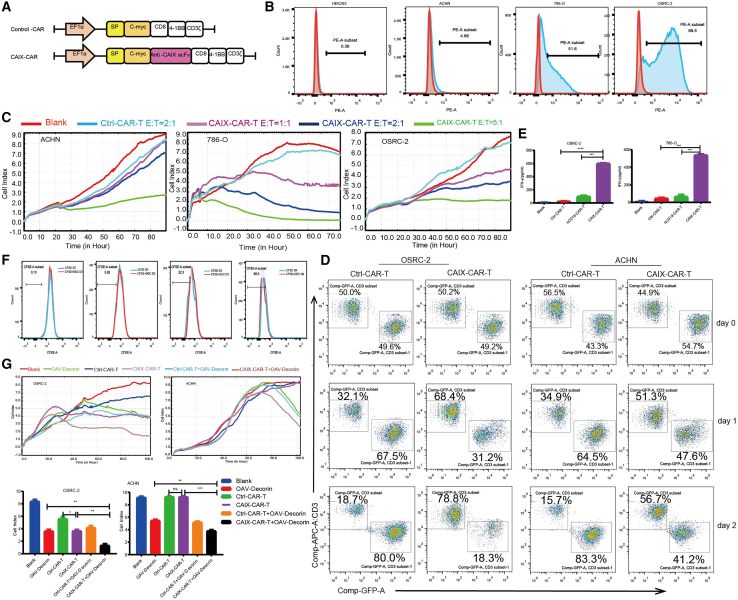
CAIX-CAR-T exerted selective cytotoxicity to CAIX-positive renal cancer cells (A) Schematic representation of CAIX-CAR. (B) Different cell lines were stained with anti-CAIX to calculate the levels of CAIX expression. (C) CAIX-CAR-T or the Control Ctrl-T cells were cocultured with ACHN, 786O, and OSRC-2 cells at the designed E:T. Viable cells were calculated as “cell index” using the xCELLigence RTCA system. (D) The killing ability of CAIX-CAR-T or Control Ctrl-T cells was also detected by flow cytometry. ACHN-GFP and OSRC-2-GFP cells were cocultured with CAR-T cells for 1 or 2 days. CAR-T cells were labeled with anti-CD3-APC. (E) Supernatants were obtained 786O or OSRC-2 after 48 h coculturing (E:T = 2:1) with CAIX-CAR-T, and the concentration of IFN-γ was analyzed by ELISA assay. (F) CAIX-CAR-T cells were pre-stained with CFSE before coculture with 50 MOI OAV-DEC and collected for flow cytometry at serial day 1, 2, 3 or 4 to monitor the results of division. (G) OSRC-2 and ACHN cells were treated with OAV-DEC (MOI = 5) following by adding Ctrl-CAR-T or Ctrl-CAR-T (E:T = 1:1). Viable cells were calculated as “cell index” using the xCELLigence RTCA system. Right panels were representative values for OSRC-2 or ACHN cells at the end of RTCA analysis. ∗p < 0.05, ∗∗p < 0.01, ∗∗∗p < 0.001, and “ns” means not significant.

Carbonic anhydrase 9 (CAIX) is a transmembrane protein and is one of only two tumor-associated carbonic anhydrase isoenzymes known. It is expressed in all clear-cell RCC, but is not detected in normal kidney or most other normal tissues.^25^ In the previous research, we constructed a CAIX-targeting CAR-T (CAIX-CAR-T), which was equipped with a CAIX-targeted CAR consisting of an scFv, a CD8α transmembrane domain, and a 4-1BB signaling intracellular domain, and together with its blank control (Ctrl-CAR-T) (Figure 2A). To measure the transduction efficiency of CAR-T cells, we inserted a c-myc tag acting as a marker for flow cytometry analysis. This CAR-T product was proven to be effective and safe in our previous research for a series of CAIX-positive tumors. The level of CAIX expression varied among the three tested types of renal cancer cell lines, with the highest on OSRC-2, moderate on 786O, and the lowest on ACHN. The normal human embryo kidney HEK293 cells also showed low level of CAIX expression (Figure 2B). To test the function of the CAIX-CAR-T, we cocultured CAIX-CAR-T cells with renal cancer cells at different effector:target (E:T) ratios of 1:1, 2:1, and 5:1. Compared with Ctrl-CAR-T (E:T = 2:1), CAIX-CAR-T presented the highest cytotoxicity efficacy for CAIX-positive renal cancer cells OSRC-2 and 786O but a least cytotoxicity for CAIX-low expression ACHN, in which CAIX-CAR-T with high E:T rate (5:1) could cause significant cytotoxicity (Figure 2C). The killing ability of CAR-T cells was also quantified by flow cytometry. Ctrl-CAR-T or CAIX-CAR-T (E:T = 2:1) cells was cocultured with OSRC-2 (CAIX-positive) or ACHN (CAIX low expression) for 24 h or 48 h. As shown in Figure 2D, CAIX-CAR-T killed most of OSRC-2 cells after 2 days, but Ctrl-CAR-T cells did not work. In ACHN cells, the killing ability of CAIX-CAR-T or Ctrl-CAR-T cells was weak. As coculture time increased, the proliferation of CAIX-CAR-T and Ctrl-CAR-T cells decreased. After 48 h coculturing with renal cancer cells 786O or OSRC-2 for CAIX-CAR-T cells, there was elevated expression of IFN-γ detected by ELISA. The cocultured Ctrl-CAR-T or HCD19-CAR-T (targeting human CD19) used as a control group did not show augmented release of IFN-γ (Figure 2E). These results showed that CAIX-CAR-T exerted selective cytotoxicity to CAIX-positive renal cancer cells.

Next, we wanted to detect the antitumor effect of oncolytic adenovirus OAV-DEC in combination with CAR-T *in vitro*. CFSE-stained CAIX-CAR-T cells were added with high MOI OAV-DEC (MOI = 50) and cultured for serial 4 days. The proliferation of CAR-T cells was analyzed with flow cytometry every day. As shown in Figure 2F, OAV-DEC had no effect on the proliferation ability of CAIX-CAR-T cells. Then OSRC-2 and ACHN cells were treated with low MOI OAV-DEC (MOI = 5) followed by adding Ctrl-CAR-T or CAIX-CAR-T (E:T = 1:1). RTCA assay results showed that Ctrl-CAR-T alone did not significantly inhibit the tumor cells, but OAV-DEC or CAIX-CAR-T treatment alone could effectively inhibit the growth of renal cancer cells with the extending of incubation time, and the inhibition effect was enhanced after the combination of the two; in addition, the difference was statistically significant compared with the control group in OSRC-2 cells with high CAIX expression. However, in ACHN cells with low expression of CAIX, cell growth was inhibited only in OAV-DEC alone and combined treatment groups (Figure 2G). These results suggested that the combination of OAV-DEC and CAIX-CAR-T can effectively suppress the growth of CIAX-positive renal carcinoma cells *in vitro* and this combination treatment modality is feasible.

### Combination of OAV-DEC and CAIX-CAR-T therapy delivered more robust antitumor effect than monotherapy *in vivo*

**Figure 3:**
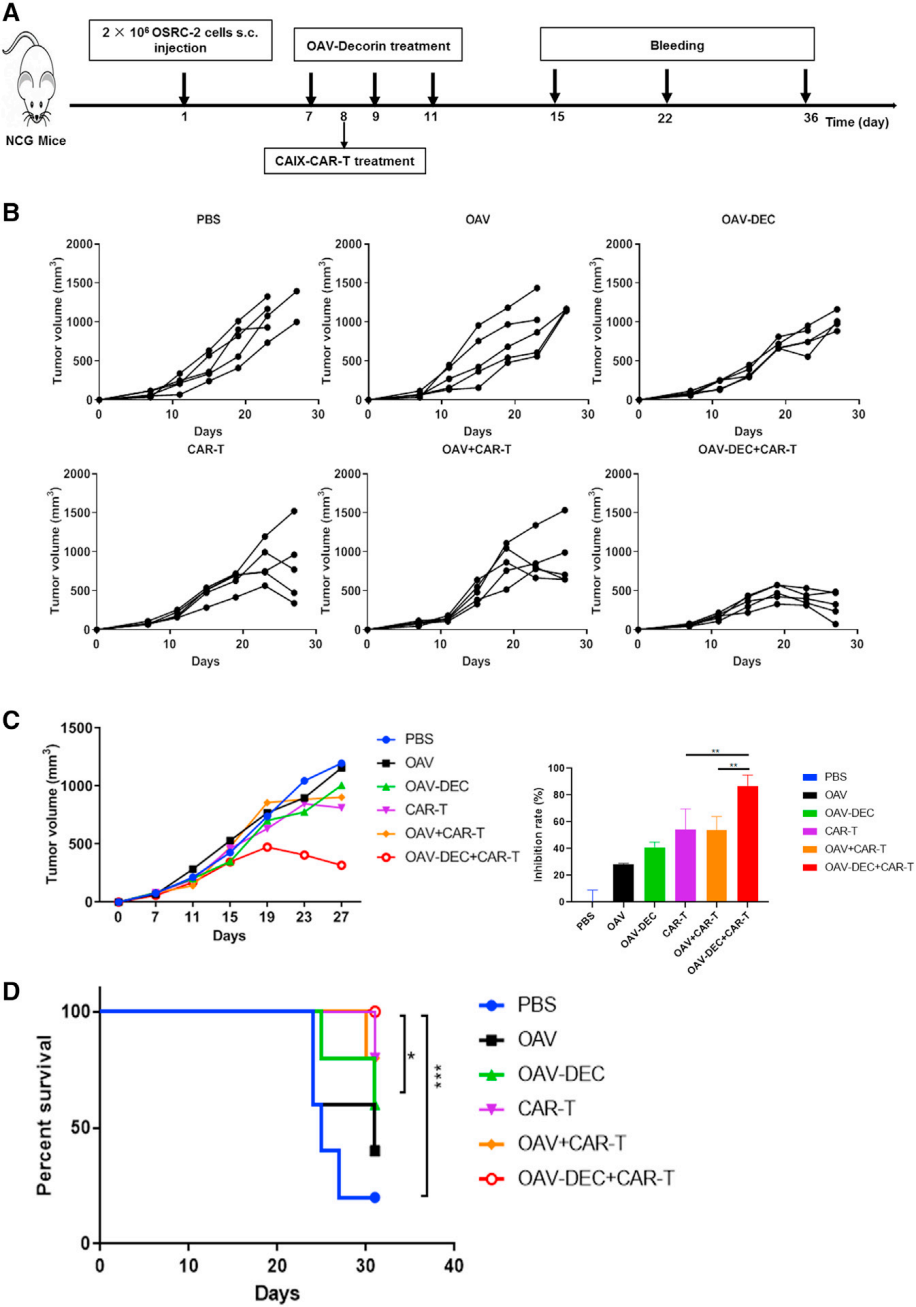
Combination of OAV-Decorin and CAIX-CAR-T cells improved the antitumor effect *in vivo* NCG mice were subcutaneously inoculated with OSRC-2 cells (n = 5 per group). (A) Experimental timeline for *in vivo* studies. (B) The tumor growth curves of each individual mouse were displayed. (C) The mean tumor volume curves ±SD were displayed and the under bar graphs showed the inhibition rate of each treatment group. (D) Kaplan-Meier survival analysis of mice after different treatments. ∗p < 0.05; ∗∗p < 0.01; ∗∗∗p < 0.001 (two-tailed paired t test).

The combination of virotherapy and immunotherapy emerged as a new promising strategy for cancer therapy. To investigate the efficacy of the combination OAV-DEC and CAIX-CAR-T cells, we established a subcutaneous xenograft model of human renal carcinoma (2×10^6^ cells per mouse) in NCG mice using the OSRC-2 cell line. When the subcutaneous tumor diameter was about 5 mm, mice were randomly divided into six groups: PBS, OAV, OAV-DEC, CAIX-CAR-T, OAV + CAIX-CAR-T, and an OAV-DEC + CAIX-CAR-T combination group (n = 5). Virus was intratumorally injected every other day for three injections and the total dose was 1 × 10^9^ PFU; 5×10^6^ CAR-T cells were infused intravenously after the second virus administration. Scheme of treatment schedule is shown in Figure 3A. Intratumoral administration of OAV, OAV-DEC, or intravenous infusion of CAIX-CAR-T monotherapy had moderate antitumor activity in the models tested, with tumor growth inhibition rates of 28.1%, 40.7%, and 54.4%, respectively (Figures 3B and 3C). The combination OAV-DEC + CAIX-CAR-T treatment group showed a significant reduction (86.9%) in tumor volume compared with saline control, but only 53.7% for OAV + CAIX-CAR-T. Moreover, the combination of OAV-DEC and CAIX-CAR-T presented significantly improved therapeutic efficacy, as the tumor-bearing mice had consistent tumor regression, and a total of five mice achieved long-term survival by the end of observation, day 32 (Figure 3D). These data suggested that combination of OAV-DEC and CAIX-CAR-T strategy enhanced the antitumor effect more than monotherapy *in vivo*.

### OAV-DEC promoted infiltration of CAIX-CAR-T cells efficiently in tumors

**Figure 4:**
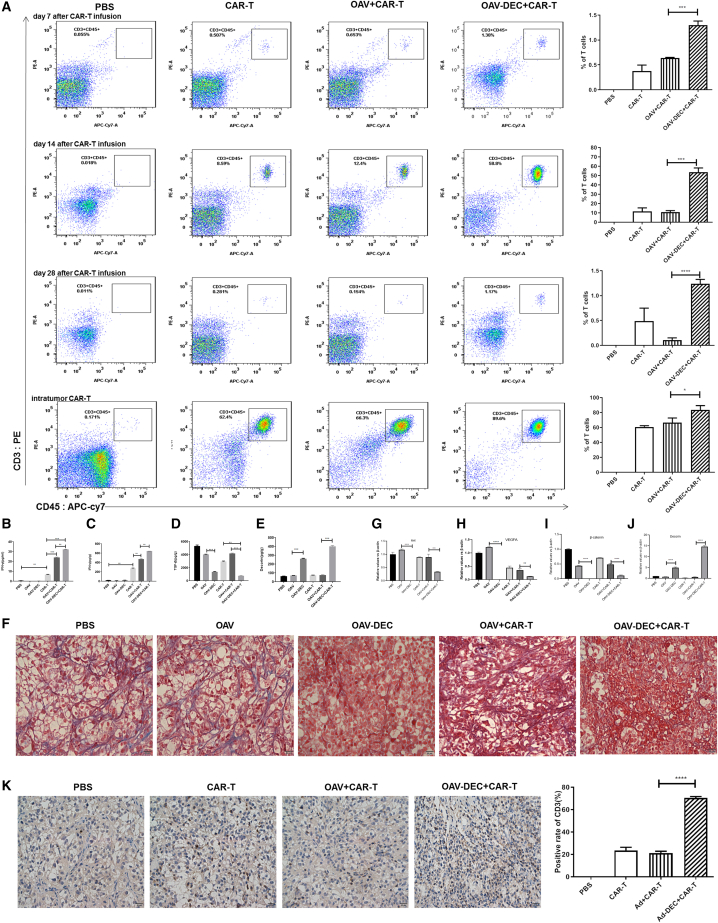
OAV-Decorin promoted infiltration of CAIX-CAR-T cells efficiently in tumors (A) The survival of CAIX-CAR-T cells in peripheral blood was detected by CD3-PE and CD45-APC-CY7 antibody on days 7, 14, and 28 after infusion by flow cytometry. The tumor tissues from two mice for each group were collected, ground, and analyzed by flow cytometry to calculate the infiltration percentages of CAR-T cells in tumor on day 14 after CAR-T infusion. At the end of the experiment, the production of IFN-γ in serum (B) or intratumor (C) from euthanized mice was analyzed by ELISA. TGF-β (D) and decorin (E) expression in tumor tissues from OAV-DEC or OAV-DEC + CAIX-CAR-T treatment were detected. (F) The expression of collagen fibers in the ECM was measured by Masson trichrome staining of extracellular matrix (blue staining) present in the tumor tissue sections. RNA was extracted from tumor tissues and the expression of Met (G), VEGFA (H), b-catenin (I), and decorin (J) was determined by quantitative PCR assay. (K) The tumors were removed from the euthanized mice, and the immune infiltrate was evaluated by immunohistochemical staining according to the distribution of CD3^+^ cells. Scale bar, 20 μm. ∗p < 0.05; ∗∗p < 0.01; ∗∗∗p < 0.001.

Aiming to gain mechanistic insight into OAV-DEC-mediated enhanced antitumor activity, we compared the presence of CAIX-CAR-T cells among the indicated groups on days 7, 14, and 28 after infusion by flow cytometry. The survival of CAIX-CAR-T cells in peripheral blood was detected by CD3-PE and CD45-APC-CY7 antibody. On day 7, a minor number of CAR-T cells were detected in the peripheral blood of the CAIX-CAR-T (0.415%), OAV + CAIX-CAR-T (0.517%), or OAV-DEC + CAIX-CAR-T (1.17%) -treated mice. On day 14, CAR-T cells increased massively in the peripheral blood of the CAIX-CAR-T (16%), OAV + CAIX-CAR-T (11.9%), and especially for the OAV-DEC + CAIX-CAR-T (58.6%) -treated mice. On day 28, CAR-T cells in the peripheral blood of mice in the combination therapy group decreased (1.25%), but were still higher than those in the CAIX-CAR-T group (0.728%). No CAR-T cells were detected in the PBS group, as expected (Figure 4A). Meanwhile, tumor tissues from two mice for each group were collected, ground, and analyzed by flow cytometry to calculate the infiltration percentages of CAR-T cells in tumor on day 14 after CAR-T infusion. The results showed that the proportion of CAR-T cells in the tumor tissues from the OAV-DEC combined with CAIX-CAR-T group was higher compared with the other groups, which was consistent with that in the peripheral blood (the last panel of Figure 4A). At the end of the experiment, we analyzed the production of IFN-γ in serum from euthanized mice. OAV-DEC + CAIX-CAR-T-treated mice had higher IFN-γ expression than that in the CAIX-CAR-T or OAV + CAIX-CAR-T group (Figure 4B). We also assessed the IFN-γ production in tumor lytic tissues. A similar result was obtained, that is, the level of IFN-γ increased in OAV-DEC + CAIX-CAR-T-treated mice (Figure 4C). Since decorin blocked the activity of TGF-β, our data also identified that OAV-DEC significantly inhibited TGF-β expression on renal cancer cells in tumor tissues from OAV-DEC or OAV-DEC + CAIX-CAR-T treatment (Figures 4D and 4E), which was identical to other research. The expression of decorin in tumor-bearing mice treated with OAV-DEC or OAV-DEC + CAIX-CAR-T evidently inhibited the distribution of collagen fibers, which is a major component of extracellular matrix (ECM) (Figure 4F). We also examined the target proteins of decorin (Figures 4G–4J), which showed that decorin production mediated by adenovirus reduced the mRNA expression of Met, b-catenin, and vascular endothelial growth factor A (VEGF-A). CD3 in tumor sections was further analyzed by immunohistochemistry analyses. As shown in Figure 4K, compared with the PBS or CAIX-CAR-T group, the OAV-DEC and OAV-DEC + CAIX-CAR-T groups showed significantly more CD3-positive staining in corresponding tumor tissues. The CD3-positive staining data showed that the mean optical density was higher in the OAV-DEC + CAIX-CAR-T combination therapy group than that in CAIX-CAR-T or OAV + CAIX-CAR-T group. There was no CD3-positive staining in the PBS, OAV, or OAV-DEC treatment groups. These data suggested that OAV-DEC did enhance the infiltration of CAIX-CAR-T in tumor tissues.

### Increased TILs after oncolytic adenovirus OAV-DEC administration in immunocompetent mouse model

**Figure 5:**
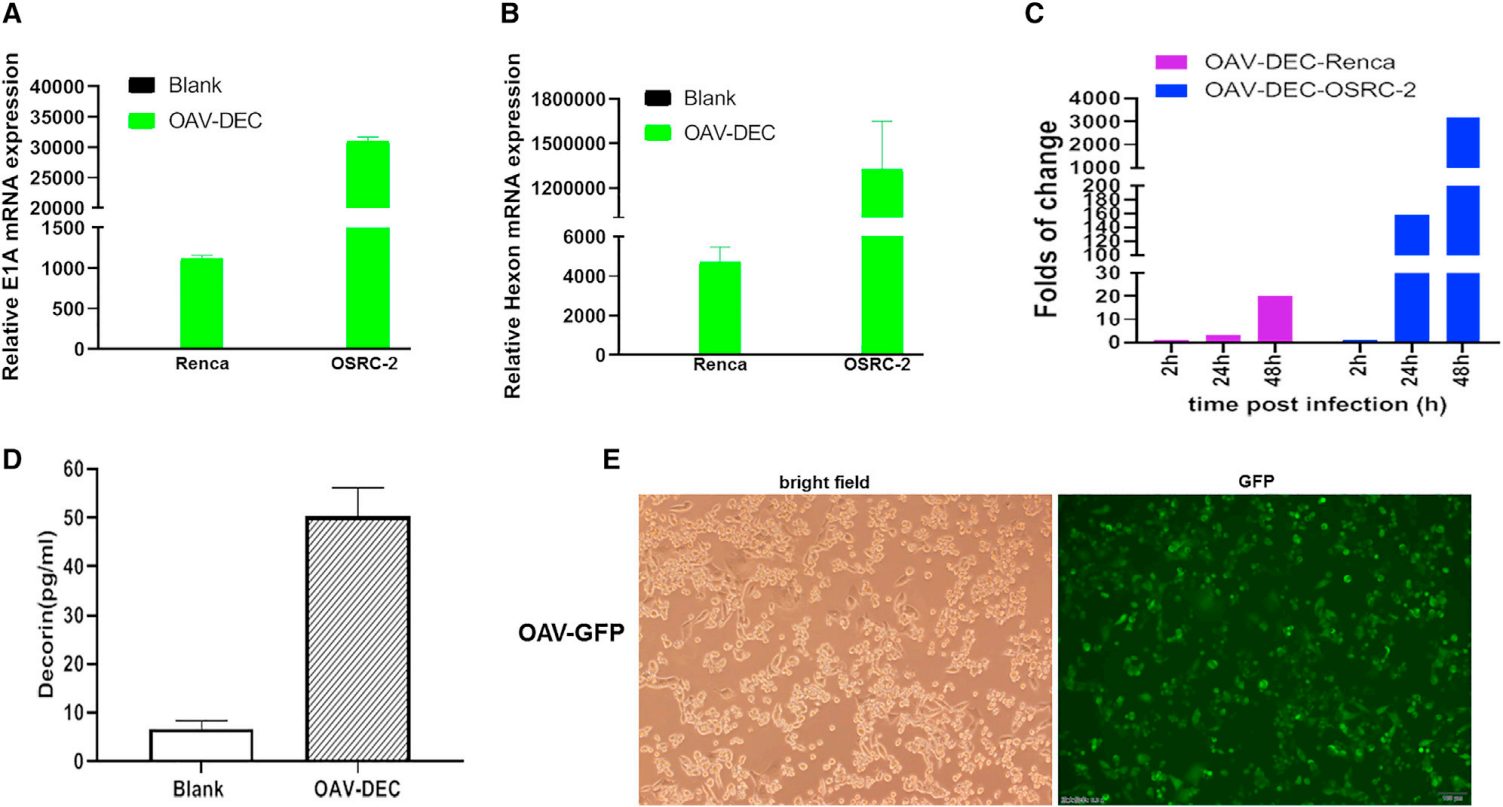
OAV-Decorin could infect and express decorin in mouse rencal cancer cells Renca (A and B) Renca and OSRC-2 cells were seeded into a six-well plate and 24 h later OAV-DEC was added into cells at MOI = 20, respectively. The adenoviral E1A (A) and Hexon (B) mRNA expression levels were analyzed by qRT-PCR. β-actin was used as control. (C) Renca and OSRC-2 cells were added 20 MOI OAV-DEC, 2 h, 24 h, and 48 h later cells were collected, frozen, and thawed three times. Viral particles in Renca and OSRC-2 were analyzed by 50% tissue culture infective dose (TCID50) in HEK293 cells. (D) Decorin expression in Renca cells added 20 MOI OAV-DEC was determined by ELISA. (E) A GFP-expressing oncolytic adenovirus OAV-GFP based on the same backbone of OAV-DEC could express GFP in Renca cells.

In order to characterize the effects of oncolytic adenovirus arming decorin (OAV-DEC) on immune response in the tumor microenvironment, we also evaluated its use in an immunocompetent mouse model because human decorin is cross-reactive for mouse cells. As reported, human adenoviruses do not replicate in most murine cancer cells, so first, we wanted to identify the actual panel of OAV-DEC in mouse renal cancer cells Renca. Oncolytic adenoviruses OAV-DEC effectively replicated in human renal cancer cells OSRC-2, but had lower replication ability in mouse renal cancer cells Renca (Figures 5A–5C). Although defective replication was detected, OAV-DEC produced high levels of decorin protein in infected Renca cells (Figure 5D). We also constructed a GFP-expressing oncolytic adenovirus OAV-GFP based on the same backbone of OAV-DEC. Renca cells added OAV-GFP could effectively display GFP expression (Figure 5E). These data indicated that OAV-DEC could infect and express the armed gene decorin although it did not replicate effectively in mouse rencal cancer cells Renca.

**Figure 6:**
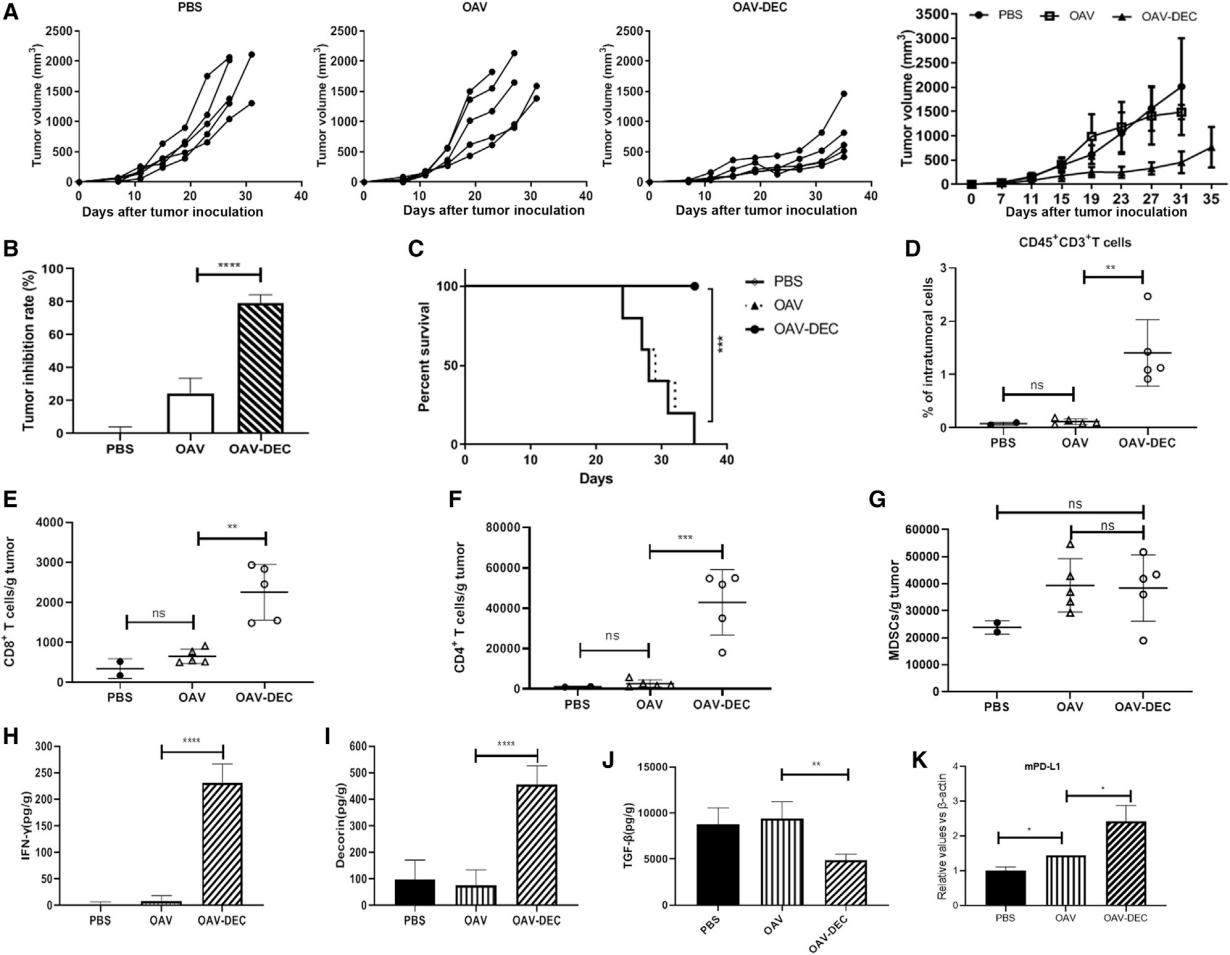
Increased TILs after oncolytic adenovirus OAV-Decorin administration in an immunocompetent mouse model (A and B) Mice were subcutaneously inoculated with Renca cells. When tumors reached about 50 mm^3^, tumors were directly injected with PBS, OAV, or OAV-DEC (1 × 10^9^ PFU) every other day, for a total of three injections. Tumor growth and the tumor inhibition rate in different groups are shown (n = 5). (C) Kaplan-Meier survival analysis of mice after different treatments. (D–G) At the end of treatment, tumors were collected and analyzed by flow cytometry to calculate the percentages of TILs in tumor cells. Percentages of CD45^+^ CD3^+^T cells (D), CD8^+^T cells (E), CD4^+^ T cells (F), and MDSC cells (G) (n = 2 to 5). (H–J) The production of mouse IFN-γ, TGF-β from euthanized mice was analyzed by ELISA. Human decorin expression mediated by OAV-DEC in tumor tissues was also detected. (K) The mRNA expression of PD-L1 in tumor tissues of each group was detected by qPCR. Mean and SD are shown for each group and compared using an independent t test. ∗p < 0.05; ∗∗p < 0.01; ∗∗∗p < 0.001; ∗∗∗∗p < 0.0001, and “ns” means not significant.

To characterize the effects of OAV-DEC on immune response, Renca cells were used to establish tumor models. Compared with the group treated with saline or OAV, intratumoral administration of OAV-DEC demonstrated antitumor activity, with tumor growth inhibition of 79.26% for the Renca cells cancer model (Figures 6A and 6B) and markedly prolonged the lifespan (Figure 6C). We compared the presence of tumor-infiltrating immune cells among the three treatment groups at 10 days in the model after the last OV treatment by flow cytometry. As we expected, intratumoral infiltration of CD45^+^CD3^+^ immune cells, CD8^+^ T cells, and CD4^+^ T cells was increased after the administration of OAV-DEC, but not for PBS or OAV. In addition, increases in the numbers of intratumoral myeloid-derived suppressor cells (MDSC) were observed, although there was no significant difference between them in OAV-DEC or OAV group (Figures 6D–6G). We especially focused on the biological activity of decorin and examined the effects of its intratumoral expression. As shown in Figures 6H–6J, intratumoral administration of OAV-DEC led to the secretion of human decorin in the injected tumors with induction of increased intratumoral murine IFN-γ protein and decreased murine TGF-β compared with OAV, indicating that intratumoral secretion of decorin promoted an inflammatory response in the tumor microenvironment. As reported, oncolytic viruses could change “cold tumors” into “hot tumors” through modulating checkpoint markers. We also examined the PD-L1 mRNA expression in OAV- or OAV-DEC-treated tumors. It showed that there was an increase level of PD-L1 in OAV- or OAV-DEC-treated tumors compared with the PBS group (Figure 6K). These results showed that intratumoral expression of decorin by an oncolytic adenovirus enhances immune response and improves tumor control.

## Discussion

In recent years, immunotherapy represented by CAR-T gradually emerges as the fourth major tumor treatment mode after surgery, radiotherapy, and chemotherapy.^7^^,^^26^ It has been widely used in clinical practice. However, the complex immunosuppressive microenvironment and physical obstacles in solid tumors, such as abnormal vasculature, increased matrix hardness, and higher tissue pressure, may impair T cell infiltration.^27^^,^^28^ This has an impact on the proliferation and persistence of T cells, greatly reducing the killing effect of CAR-T. Therefore, the key to CAR-T therapy for solid tumors is to reverse this tumor immunosuppressive microenvironment, remove the inhibition of T cell function, reduce the physical barrier, and promote its infiltration in tumor cells, so that it can obtain a better antitumor activity.^29^

In this study, we constructed an oncolytic adenovirus arming decorin and CAR-T cells targeting human CAIX. The synergistic antitumor effect of OAV-Decorin combined with CAIX-CAR-T was further investigated. *In vitro*, we found that OAV-Decorin combined with CAIX-CAR-T has a strong antitumor effect on renal cancer cells. Moreover, in the subcutaneous tumor transplantation model of NCG mice, we found that the combined treatment of CAR-T and OAV-Decorin could effectively inhibit tumor growth and greatly improve the survival percentage of the mice. OAV-Decorin or CAR-T monotherapy group had limited antitumor activity. Like other anticancer therapies, oncolytic virotherapy has several limitations, such as viral delivery to the target, penetration into the tumor mass, and antiviral immune responses.^30^ The potential of oncolytic virotherapy combined with other treatments could form part of future multimodality treatment strategies.

The CAR-T cells were detected in the peripheral blood samples taken from the tail vein of mice on days 7, 14, and 28 after infusion. The second collected blood sample assay showed that 16% was detected for CAR-T monotherapy and 58.6% in the combination group. The CAR-T content decreased on day 28 after infusion. It might be attributed to the depressed tumor. Moreover, at the end of the *in vivo* experiment, the single-cell suspension was filtered and collected by tumor homogenate, and the intratumor CAR-T cells were also detected by flow cytometry. Compared with PBS, OAV-Decorin, and CAIX-CAR-T groups, the OAV-Decorin combined with CAIX-CAR-T group had more CAR-T cell infiltration, which was consistent with blood test results. ELISA results showed that the expression of IFN-γ in OAV-Decorin combined with CAIX-CAR-T was significantly higher than that in the CAIX-CAR-T-treated group. The expression trend of IFN-γ in mice tumors was also consistent with that in serum. It showed that TGF-β level was lower than that in both the OAV-Decorin alone group and the combined group. Masson staining showed that the amount of collagen fiber in OAV-Decorin and OAV-Decorin + CAIX-CAR-T group was significantly decreased compared with PBS and CAIX-CAR-T group. CD3 and decorin expression were significantly upregulated in OAV-Decorin combined with CAIX-CAR-T group, confirming that OAV-Decorin can increase the invasion effect of CAR-T cells in tumors.

Tumor-infiltrating lymphocytes (TILs) are one of the crucial players in the TME of cancers.^31^ Suppressive immune cells, which could block antitumor responses via inhibiting effector cells, such as CD8^+^ T cells, natural killer cells, and antigen-presenting cells, are generally detected in TME and are associated with the poor prognosis of cancer.^32^ Here we found that in the antitumor activity of OAV-Decorin in immunocompetent mouse, there were increased intratumoral murine IFN-γ proteins and decreased murine TGF-β in OAV-Decorin treated-tumor compared with OAV. Moreover, intratumoral infiltration of CD45^+^CD3^+^ immune cells, CD8^+^ T cells, and CD4^+^ T cells was increased after the administration of OAV-Decorin, but not for PBS or OAV. It indicated that intratumoral secretion of decorin mediated by oncolytic adenovirus promoted an inflammatory response in the tumor microenvironment and improved tumor control in an immunocompetent mouse model.

In summary, the CAIX-CAR-T and OAV-Decorin that we constructed proved to have significant specific killing effect on CAIX-positive renal cancer cells *in vitro*, and the combination usage can further enhance the tumor killing effect. *In vivo* experiments further confirmed that OAV-Decorin can change the composition of ECM in renal cancer tissues, inhibit the expression of TGF-β, increase the infiltration of OAV and CAR-T in tumor tissues, and reduce the inhibition of CAR-T cells in the immunosuppressive microenvironment, thus achieving the antitumor effect. We found that OAV-Decorin or CAR-T cells alone did not have significant efficiency *in vivo*. We considered it might be because NCG mice do not have immunity, so the local inflammation caused by oncolytic viruses cannot induce the host's antitumor immune response. The poor efficacy of CAR-T therapy alone may be due to the complex tumor microenvironment, the inability of CAR-T to penetrate its physical barrier, and the poor proliferation in tumor and other mechanisms, which need to be further studied.

To conclude, we have shown that the combined use of OAV-Decorin and CAIX-CAR-T displayed synergistic antitumor effects *in vitro* and *in vivo* by enhancing T cell persistence, which led to prolonged survival of the tumor-bearing mice. These data laid a foundation for the further clinical study of the combination therapy for RCC.

## Materials and methods

### Cell lines and viruses

The murine renal cancer cell lines Renca, human embryonic kidney (HEK)-293, human renal carcinoma 786-O, ACHN, and OSRC-2 were obtained from the American Type Culture Collection (Manassas, VA) and the RIKEN (Tokyo, Japan), respectively. HEK293, ACHN, and OSRC-2 cells were maintained in Dulbecco's modified Eagle's medium. Renca and 786-O were maintained in RPMI1640 medium. ACHN, OSRC-2 cells stably expressing GFP were constructed and generated by a GFP lentiviral. All media were supplemented with 10% fetal bovine serum, 100 U/mL penicillin, and 100 mg/mL streptomycin. Cells were not cultured for more than 5 months following resuscitation especially for HEK293 cells. These cells were maintained at 37°C in a humidified atmosphere with 5% CO_2_.

An engineered oncolytic adenovirus expressing decorin named OAV-Decorin (OAV-DEC) was constructed previously,^33^ in the backbone of a tumor-selective oncolytic adenovirus ZD55 vector, in which E1B-55KD had been deleted. HEK293 cells were used to amplify virus OAV-DEC or control vector virus OAV with a density of about 80%, when half of the cells were floated, the viruses were collected and the titers were measured by tissue culture infection dose 50 (TCID50) methods.^34^

### Primary T cell isolation, culture, and transduction

Peripheral blood mononuclear cells from healthy donors were collected and activated by human T-activator CD3/CD28 Dynabeads (Gibco, Life Technologies) as the manufacturer's protocol. T cells were cultured in X-vivo 15 (LONZA, Walkersville, MD) supplemented with 1% human serum albumin, 1% P/S, and 200U IL-2 (PrimeGene, China). The medium was replaced every other day. Forty-eight hours post activation, T cells were transduced with the lentivirus at an MOI of 10 with spin infection (1800 rpm, 1.5 h). The lentivirus expressing CAIX-specific CAR was constructed in our laboratory. Three days after infection, T cells were analyzed for CAR^+^ population percentage by flow cytometry.

### Western blot

Proteins were extracted from the cells. An aliquot of 100 μg total protein was used SDS-PAGE gel and transferred to a PVDF membrane. Membranes were incubated with blocking buffer (5% nonfat milk in TBS containing 0.1% Tween 20) for 2 h at room temperature. Thereafter, samples were probed overnight at 4°C with primary antibodies against E1A (Merk Millipore) or GAPDH (Santa Cruz). After extensive washing, the membrane was incubated with secondary antibody immunoglobulin G (Zhongshan, Beijing) for 1 h at room temperature. The blotting membranes were visualized by a Bio-Rad chemiluminometer.

### ELISA experiment

Cells (3×10^5^ in a six-well plate) were infected with OAV-DEC at MOI = 20 for 24 h. The culture medium was removed, and the 800 μL serum-free culture medium was added for 24 h. Then, the supernatant was collected. The protein expression was determined by ELISA according to the manufacturer's protocol.

### CCK-8 proliferation experiment

Inoculated cells were infected with OAV-DEC at MOI = 1, 5, 20, or 50. Four days after infection, cell proliferation was analyzed by recording the absorbance of the sample at 450 nm using the CCK-8 assay. The survival rate was calculated: survival rate = (experimental well-blank well)/(control well-blank well) ×100%. The final data were the average of three independent experiments.

### xCELLigence

The xCELLigence RTCA system instrument (ACEA Biosciences) was used for impedance experiments to determine tumor cell killing according to the manufacturer's protocol. Briefly, tumor cells were plated at 10,000 cells per well, 12 h later, following by adding MOIs of OAV-DEC or CAR-T cells with different effector: target ratios. For CAR-T cells–mediated tumor killing or combination with OAV-DEC assay, CAR-T cells and the indicated MOI (MOI = 5) of OAV-DEC were added to the cells at the same time.

### Flow cytometric analysis

To analyze the expression of CAIX in different cell lines, cells were incubated with antibody to human CAIX PE (Invitrogen). Intratumoral cells of mice were stained with the following antibodies: mouse CD3 fluorescein isothiocyanate (FITC) (BD Biosciences), mouse CD4 phycoerythrin (PE), mouse CD8 allophycocyanin (APC), mouse CD45 PE–Cy7, mouse CD3 PE, mouse CD4 FITC, mouse CD25 APC-Cy7, mouse CD3 PE. Human CAR-T cells obtained from the CAR-T-infused NCG mice PBMC or tumors were stained with the following antibodies: human CD45 APC-Cy7, human CD3 PE. Samples were also analyzed on a BD FACS Canto II flow cytometer (Becton Dickinson). Data were analyzed by FlowJo (Tree Star Inc.).

### CFSE cell labeling

To detect the effect of oncolytic adenovirus OAV-DEC on CAR-T cell proliferation, CAIX-CAR-T cells were labeled with CFSE at a final concentration of 10 μM and then 50 MOI OAV-DEC. After 24 h, 48 h, 72 h, and 96 h, CAIX-CAR-T cells were collected, and the proliferation of CAIX-CAR-T cells was detected by flow cytometry.

### RNA isolation, reverse transcription, and quantitative real-time PCR

OSRC-2 and Renca cells were plated in six-well plates and for different experiments; 50 mg tumor tissues from different treatment groups were also collected. Total cellular RNA was extracted using 1 mL of TRIzol reagent (Invitrogen) per well in 6-well plates; 500 ng RNA was used for cDNA synthesis with HiScript Reverse Transcriptase reagent Kit (Vazyme biotech, China). Samples were analyzed in triplicate by the Applied Biosystems 7500 PCR System. The targeted genes and endogenous housekeeping gene β-actin were used as normalizing controls and amplified, using the PrimeScript RT reagent Kit (TaKaRa). The cycle number (Ct) was calculated and the fold changes of gene expression were determined using the double ΔCt (2-ΔΔCT) method.

### *In vivo* tumor studies

All animal experiments were performed under protocols approved by Xuzhou Medical University Institutional Animal Care and Use Committee. For human tumor xenograft studies, OSRC-2 cells (2 × 10^6^ cells per mouse) were prepared and injected subcutaneously into the right flank of male NCG mice (purchased from Nanjing University Model Animal Institute, China). Tumor growth and mice body weight were monitored two to three times per week by caliper measurement. Tumor volume was calculated by the formula length×width^2^×0.5. Once tumor volumes reached about 50 to 100 mm^3^, PBS, oncolytic adenovirus OAV, or OAV-DEC was intratumorally administered every other day for a total of three injections at 10^9^ PFU per mouse. For combination therapy or CAR-T monotherapy, CAIX-CAR-T cells (5×10^6^ cells per mouse) were injected intratumorally on the next days after the second OAV or OAV-DEC treatment. For CAIX-CAR-T cell detection in blood, bleeding was performed on day 7, 14, or 28 after CAR-T infusion from three mice for each group. For all studies, mice were euthanized, and tumors were harvested and processed for flow cytometry once tumors reached no more than 15 mm in diameter. For measurement of intratumoral cytokines, tumor samples were collected and immediately frozen in liquid nitrogen. Intratumoral or serum human IFN-γ was quantified using human IFN-γ ELISA Kit (Dakewe Biotech, China). Intratumoral human TGF-β and decorin were quantified using human TGF-β (Dakewe Biotech, China) and Decorin ELISA Kit (Abcam) respectively.

For subcutaneously immunocompetent mouse studies, mouse rencal cancer cells Renca cells (5×10^5^ cells per mouse) were engrafted in the right flank of male BALB/c mice by subcutaneous injection. Tumor growth was monitored two to three times per week by caliper measurement. Once tumor volumes reached about 50 to 100 mm^3^, PBS, OAV, or OAV-DEC were intratumorally administered for a total of 10^9^ PFU per mouse. Tumor growth was monitored two to three times per week by caliper measurement. Ten days after the last treatment, tumors from two mice for each group were collected and analyzed by flow cytometry to calculate the percentages of TILs in tumor cells.

### Immunohistochemistry staining analysis

The tumor tissue were fixed in 10% formalin, embedded in paraffin. and cut into 3-mm sections. Deparaffinized tumor sections were treated with primary antibody for decorin, CD3, or Collagen Type I. After incubation with a secondary antibody, tissue sections were then counterstained with hematoxylin. Masson trichrome staining was used to analyze the distribution of collagen fibers (stained blue color). Representative sections were stained with Masson trichrome and then observed by light microscopy. The collagen fiber area was analyzed with Image-Pro Plus software.

### Statistical analysis

Statistical analysis was conducted with GraphPad Prism 8. Data are presented as means ± SD. Statistical comparisons between groups were performed using the unpaired two-tailed Student t test to calculate p value. The survival curve was obtained by Kaplan-Meier plot, and a two-sided log rank test was applied for mouse survival test; p < 0.05 was considered as significant (∗p < 0.05; ∗∗p < 0.01; ∗∗∗p < 0.001).
